# 3,3′-Carbonyl­dipyridinium bis­(perchlorate)

**DOI:** 10.1107/S1600536812022817

**Published:** 2012-05-26

**Authors:** Ya Zhang, Ai-min Li, Zhi-wei Wang, Chong-Qing Wan

**Affiliations:** a Department of Chemistry, Capital Normal University, Beijing 100048, People’s Republic of China

## Abstract

In the title molecular salt, C_11_H_10_N_2_O^2+^·2ClO_4_^−^, the complete cation is generated by crystallographic twofold symmetry. The dihedral angle between the pyridyl rings is 67.07 (7)°. The crystal structure features N—H⋯Cl hydrogen bonds, forming sheets in the *ab* plane.

## Related literature

For the dipyridyl ketone dication, see: Crook & McElvain (1930 ▶); Favaro *et al.* (1990 ▶). For metal complexes of di-3-pyridyl ketone, see: Chen & Mak (2005 ▶); Chen *et al.* (2009 ▶).
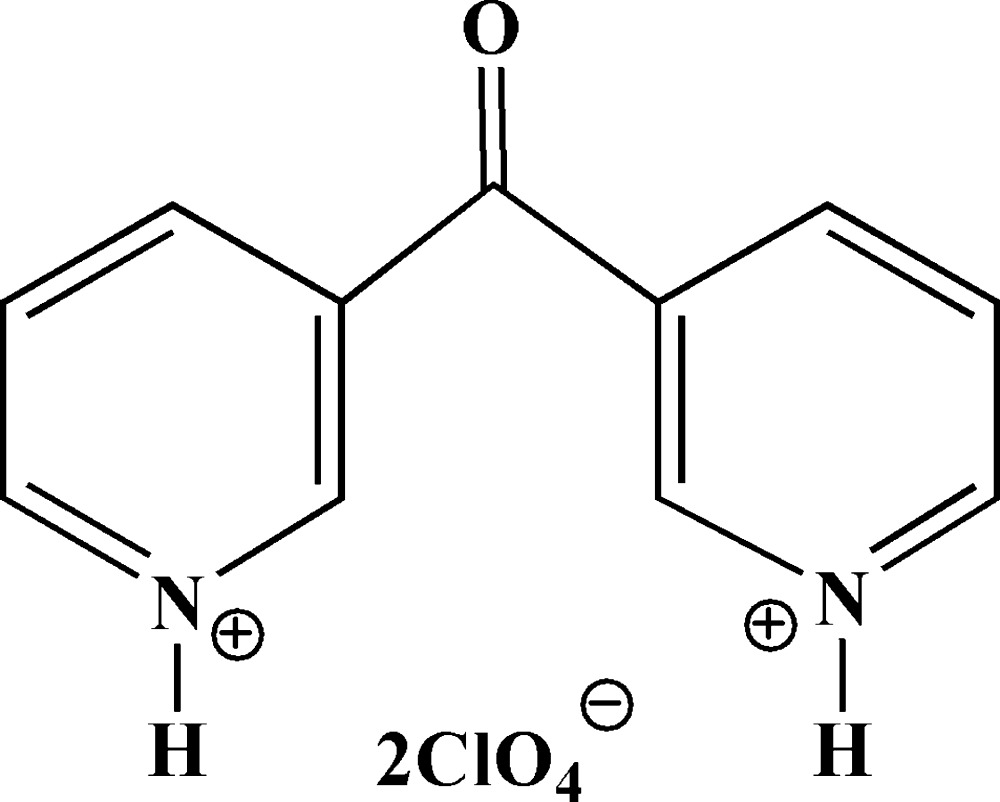


## Experimental

### 

#### Crystal data

C_11_H_10_N_2_O^2+^·2ClO_4_^−^
*M* *_r_* = 385.11
Orthorhombic, 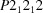

*a* = 8.5315 (3) Å
*b* = 15.1772 (6) Å
*c* = 5.6107 (2) Å
*V* = 726.50 (5) Å^3^
*Z* = 2
Mo *K*α radiation
μ = 0.50 mm^−1^
*T* = 296 K
0.40 × 0.30 × 0.20 mm

#### Data collection

Bruker APEXII CCD area-detector diffractometer
Absorption correction: multi-scan (*SADABS*; Bruker, 2007 ▶) *T*_min_ = 0.835, *T*_max_ = 0.905
6343 measured reflections
1285 independent reflections
1235 reflections with *I* > 2σ(*I*)
*R*_int_ = 0.021

#### Refinement

*R*[*F* ^2^ > 2σ(*F* ^2^)] = 0.028
*wR*(*F* ^2^) = 0.073
*S* = 1.10
1285 reflections
111 parameters
H-atom parameters constrained
Δρ_max_ = 0.34 e Å^−3^
Δρ_min_ = −0.21 e Å^−3^
Absolute structure: Flack (1983 ▶), 503 Friedel pairs
Flack parameter: 0.10 (10)

### 

Data collection: *APEX2* (Bruker, 2007 ▶); cell refinement: *APEX2* and *SAINT* (Bruker, 2007 ▶); data reduction: *SAINT*; program(s) used to solve structure: *SHELXS97* (Sheldrick, 2008 ▶); program(s) used to refine structure: *SHELXL97* (Sheldrick, 2008 ▶); molecular graphics: *SHELXTL* (Sheldrick, 2008 ▶); software used to prepare material for publication: *SHELXTL* and *PLATON* (Spek, 2009 ▶).

## Supplementary Material

Crystal structure: contains datablock(s) I, global. DOI: 10.1107/S1600536812022817/bt5923sup1.cif

Structure factors: contains datablock(s) I. DOI: 10.1107/S1600536812022817/bt5923Isup2.hkl

Supplementary material file. DOI: 10.1107/S1600536812022817/bt5923Isup3.cml

Additional supplementary materials:  crystallographic information; 3D view; checkCIF report

## Figures and Tables

**Table 1:** Hydrogen-bond geometry (Å, °)

*D*—H⋯*A*	*D*—H	H⋯*A*	*D*⋯*A*	*D*—H⋯*A*
N1—H7⋯O2	0.86	2.22	2.907 (3)	136
N1^i^—H7^i^⋯O4	0.86	2.34	2.967 (2)	130

Symmetry code: (i) 
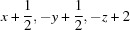
.

**Fig. 1.**
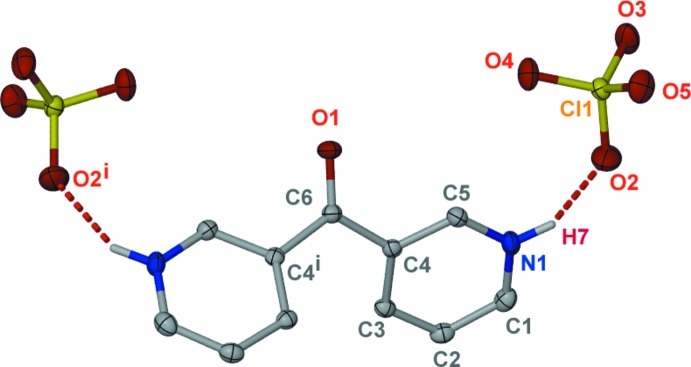
The atom-numbering scheme of the title salt; some H atoms have been omitted for clarity. Displacement ellipsoids are shown at the 30% probability level. [Symmetry code: (i) -x + 1, -y, z.]

## supplementary crystallographic information

### Comment

Di-3-pyridyl ketone is an extraordinary ligand within the family of basic building blocks for construction of metal-organic complexes with intriguing architectures (Chen & Mak, 2005; Chen *et al.*, 2009). However, the crystal structure of salts with the dipyridyl ketone dication is rarely reported until now. Several related literatures discussed the relationship between the acid-base properties of the dipyridyl ketone isomers and the positions of the nitrogen atoms on the rings, which were investigated by spectrophotometric measurements (Crook & McElvain, 1930; Favaro *et al.*, 1990). In the present context, we report the structure of diprotonated di-3-pyridyl ketone perchlorate salt (Fig. 1). The two pyridyl rings exhibit a dihedral angle of 67.07 (7)°. The crystal structure is stabilized by N—H···(perchlorate) hydrogen bonds forming sheets in the ab plane.

### Experimental

Di-3-pyridyl ketone was prepared following the literature procedure of Chen & Mak (2005). Copper(II) perchlorate (37 mg, 0.1 mmol) was heated with di-3-pyridyl ketone (18 mg, 0.1 mmol) in acetonitrile (5 ml) at 373 K for 24 h. After cooling to room temperature, the precipitate which had formed was filtrated off. Crystals of the title salt was deposited by slow evaporation of the filtrate, which can be viewed as the product of the perchloric acid from the copper(II) perchlorate and di-3-pyridyl ketone (yield 11.5 mg, 30% based on di-3-pyridyl ketone).

### Refinement

H atoms were placed in idealized positions and allowed to ride on their parent atoms, with C—H = 0.93 Å and N—H = 0.86 Å, and with *U*_iso_(H) = 1.2*U*_eq_(C,N).

### Crystal data

**Table:**

C_11_H_10_N_2_O^2+^·2ClO_4_^−^	*D*_x_ = 1.760 Mg m^−^^3^
*M**_r_* = 385.11	Mo *K*α radiation, λ = 0.71073 Å
Orthorhombic, *P*2_1_2_1_2	Cell parameters from 256 reflections
*a* = 8.5315 (3) Å	θ = 2.3–26.2°
*b* = 15.1772 (6) Å	µ = 0.50 mm^−^^1^
*c* = 5.6107 (2) Å	*T* = 296 K
*V* = 726.50 (5) Å^3^	Block, colorless
*Z* = 2	0.40 × 0.30 × 0.20 mm
*F*(000) = 392	

### Data collection

**Table:**

Bruker APEXII CCD area-detector diffractometer	1285 independent reflections
Radiation source: fine-focus sealed tube	1235 reflections with *I* > 2σ(*I*)
Graphite monochromator	*R*_int_ = 0.021
ω scans	θ_max_ = 25.0°, θ_min_ = 2.7°
Absorption correction: multi-scan (*SADABS*; Bruker, 2007)	*h* = −10→10
*T*_min_ = 0.835, *T*_max_ = 0.905	*k* = −18→16
6343 measured reflections	*l* = −6→6

### Refinement

**Table:**

Refinement on *F*^2^	Hydrogen site location: inferred from neighbouring sites
Least-squares matrix: full	H-atom parameters constrained
*R*[*F*^2^ > 2σ(*F*^2^)] = 0.028	*w* = 1/[σ^2^(*F*_o_^2^) + (0.0315*P*)^2^ + 0.4015*P*] where *P* = (*F*_o_^2^ + 2*F*_c_^2^)/3
*wR*(*F*^2^) = 0.073	(Δ/σ)_max_ < 0.001
*S* = 1.10	Δρ_max_ = 0.34 e Å^−^^3^
1285 reflections	Δρ_min_ = −0.21 e Å^−^^3^
111 parameters	Extinction correction: *SHELXL97* (Sheldrick, 2008), Fc^*^=kFc[1+0.001xFc^2^λ^3^/sin(2θ)]^-1/4^
0 restraints	Extinction coefficient: 0.019 (3)
Primary atom site location: structure-invariant direct methods	Absolute structure: Flack (1983), 503 Friedel pairs
Secondary atom site location: difference Fourier map	Flack parameter: 0.10 (10)

### Special details

**Table:**

Geometry. All esds (except the esd in the dihedral angle between two l.s. planes) are estimated using the full covariance matrix. The cell esds are taken into account individually in the estimation of esds in distances, angles and torsion angles; correlations between esds in cell parameters are only used when they are defined by crystal symmetry. An approximate (isotropic) treatment of cell esds is used for estimating esds involving l.s. planes.
Refinement. Refinement of *F*^2^ against ALL reflections. The weighted *R*-factor *wR* and goodness of fit *S* are based on *F*^2^, conventional *R*-factors *R* are based on *F*, with *F* set to zero for negative *F*^2^. The threshold expression of *F*^2^ > 2σ(*F*^2^) is used only for calculating *R*-factors(gt) *etc*. and is not relevant to the choice of reflections for refinement. *R*-factors based on *F*^2^ are statistically about twice as large as those based on *F*, and *R*- factors based on ALL data will be even larger.

### Fractional atomic coordinates and isotropic or equivalent isotropic displacement parameters (Å^2^)

**Table:**

	*x*	*y*	*z*	*U*_iso_*/*U*_eq_	
C1	0.1257 (3)	0.13417 (17)	0.4051 (5)	0.0416 (6)	
H1	0.0408	0.1642	0.3402	0.050*	
C2	0.1807 (3)	0.05924 (17)	0.2988 (4)	0.0372 (6)	
H2	0.1340	0.0381	0.1604	0.045*	
C3	0.3062 (3)	0.01520 (14)	0.3985 (4)	0.0317 (5)	
H3	0.3449	−0.0357	0.3274	0.038*	
C4	0.3745 (3)	0.04756 (14)	0.6063 (4)	0.0287 (5)	
C5	0.3158 (3)	0.12400 (15)	0.7048 (4)	0.0334 (5)	
H5	0.3609	0.1474	0.8419	0.040*	
C6	0.5000	0.0000	0.7390 (6)	0.0308 (7)	
N1	0.1948 (2)	0.16399 (13)	0.6032 (4)	0.0386 (5)	
H7	0.1591	0.2113	0.6677	0.046*	
O1	0.5000	0.0000	0.9548 (4)	0.0431 (6)	
Cl1	0.30796 (6)	0.32979 (4)	1.13244 (10)	0.03518 (19)	
O2	0.2552 (3)	0.31908 (15)	0.8930 (4)	0.0720 (7)	
O3	0.1779 (3)	0.33349 (15)	1.2909 (4)	0.0647 (6)	
O4	0.4064 (2)	0.25628 (13)	1.1946 (4)	0.0539 (6)	
O5	0.3940 (3)	0.41004 (13)	1.1460 (5)	0.0622 (6)	

### Atomic displacement parameters (Å^2^)

**Table:**

	*U*^11^	*U*^22^	*U*^33^	*U*^12^	*U*^13^	*U*^23^
C1	0.0318 (12)	0.0470 (14)	0.0461 (16)	0.0021 (11)	−0.0001 (12)	0.0095 (13)
C2	0.0350 (13)	0.0444 (14)	0.0323 (12)	−0.0082 (12)	−0.0038 (11)	0.0022 (10)
C3	0.0339 (11)	0.0321 (12)	0.0293 (11)	−0.0041 (10)	0.0037 (12)	−0.0010 (10)
C4	0.0293 (11)	0.0299 (11)	0.0269 (11)	−0.0019 (9)	0.0043 (11)	0.0025 (10)
C5	0.0348 (12)	0.0336 (12)	0.0319 (12)	−0.0013 (11)	0.0021 (11)	−0.0022 (9)
C6	0.0346 (18)	0.0289 (17)	0.0288 (18)	−0.0017 (15)	0.000	0.000
N1	0.0378 (10)	0.0327 (10)	0.0452 (12)	0.0081 (10)	0.0046 (10)	−0.0007 (10)
O1	0.0499 (15)	0.0537 (16)	0.0259 (13)	0.0073 (14)	0.000	0.000
Cl1	0.0335 (3)	0.0328 (3)	0.0393 (3)	−0.0005 (2)	0.0001 (3)	−0.0034 (3)
O2	0.0990 (17)	0.0660 (14)	0.0511 (12)	0.0026 (13)	−0.0214 (12)	−0.0129 (12)
O3	0.0620 (13)	0.0551 (12)	0.0770 (15)	0.0139 (12)	0.0314 (11)	0.0109 (12)
O4	0.0398 (10)	0.0402 (10)	0.0817 (16)	0.0070 (8)	−0.0062 (10)	−0.0008 (10)
O5	0.0604 (12)	0.0403 (11)	0.0858 (16)	−0.0138 (9)	−0.0075 (14)	0.0029 (12)

### Geometric parameters (Å, º)

**Table:**

C1—N1	1.337 (3)	C5—N1	1.327 (3)
C1—C2	1.368 (4)	C5—H5	0.9300
C1—H1	0.9300	C6—O1	1.210 (4)
C2—C3	1.381 (4)	C6—C4^i^	1.491 (3)
C2—H2	0.9300	N1—H7	0.8600
C3—C4	1.393 (3)	Cl1—O3	1.423 (2)
C3—H3	0.9300	Cl1—O5	1.4242 (19)
C4—C5	1.379 (3)	Cl1—O2	1.426 (2)
C4—C6	1.491 (3)	Cl1—O4	1.439 (2)
			
N1—C1—C2	119.5 (2)	C4—C5—H5	120.2
N1—C1—H1	120.2	O1—C6—C4	119.97 (14)
C2—C1—H1	120.2	O1—C6—C4^i^	119.97 (14)
C1—C2—C3	119.5 (2)	C4—C6—C4^i^	120.1 (3)
C1—C2—H2	120.3	C5—N1—C1	123.1 (2)
C3—C2—H2	120.3	C5—N1—H7	118.5
C2—C3—C4	119.5 (2)	C1—N1—H7	118.5
C2—C3—H3	120.2	O3—Cl1—O5	109.56 (14)
C4—C3—H3	120.2	O3—Cl1—O2	110.31 (16)
C5—C4—C3	118.7 (2)	O5—Cl1—O2	108.08 (15)
C5—C4—C6	117.9 (2)	O3—Cl1—O4	109.53 (13)
C3—C4—C6	123.2 (2)	O5—Cl1—O4	110.44 (12)
N1—C5—C4	119.7 (2)	O2—Cl1—O4	108.90 (14)
N1—C5—H5	120.2		
			
N1—C1—C2—C3	0.4 (4)	C5—C4—C6—O1	34.0 (2)
C1—C2—C3—C4	0.2 (3)	C3—C4—C6—O1	−141.03 (17)
C2—C3—C4—C5	−1.0 (3)	C5—C4—C6—C4^i^	−146.0 (2)
C2—C3—C4—C6	174.0 (2)	C3—C4—C6—C4^i^	38.97 (17)
C3—C4—C5—N1	1.2 (3)	C4—C5—N1—C1	−0.6 (4)
C6—C4—C5—N1	−174.1 (2)	C2—C1—N1—C5	−0.2 (4)

Symmetry code: (i) −*x*+1, −*y*, *z*.

### Hydrogen-bond geometry (Å, º)

**Table:**

*D*—H···*A*	*D*—H	H···*A*	*D*···*A*	*D*—H···*A*
N1—H7···O2	0.86	2.22	2.907 (3)	136
N1^ii^—H7^ii^···O4	0.86	2.34	2.967 (2)	130

Symmetry code: (ii) *x*+1/2, −*y*+1/2, −*z*+2.
